# Special Issue “COVID-19 and Venous Thromboembolism”

**DOI:** 10.3390/jcm11133822

**Published:** 2022-07-01

**Authors:** Luca Costanzo

**Affiliations:** Unit of Angiology, Department of Cardio-Thoraco-Vascular, Policlinico “G. Rodolico—San Marco” University Hospital, University of Catania, 95100 Catania, Italy; lucacost84@gmail.com

In the last two years, the new coronavirus has afflicted the whole world causing a pandemic burdened by high morbidity and mortality [1]. Over recent months, increasing evidence has been brought to light that venous thromboembolism (VTE) was a frequent complication that adversely affected the clinical course of the coronavirus disease 2019 (COVID-19), especially in the most severe cases [2]. Consequently, numerous researchers around the world have focused efforts on identifying the underlying etiopathogenetic mechanisms of vascular involvement in COVID-19 and on potential therapies that can improve the outcome on these patients. The present Special Issue in the *Journal of Clinical Medicine* (JCM) is dedicated to COVID-19 and VTE, and high-quality scientific papers have been selected with the aim of implementing the scientific evidence on this topic.

In the first paper, Trimaille et al. [3] investigated the determinants and prognosis of acute pulmonary embolism (APE) during COVID-19. Among a total of 140 patients, authors noticed a decline in the non-COVID-19 incidence of APE. Additionally, although the characteristics of thrombus load and location did not differ among COVID-19 and non-COVID-19 patients, an increase of markers of coagulation and inflammation together with a worse outcome were observed in COVID-19 patients with APE. These results are in line with previous reports that emphasized the concept of immune-thrombosis in such subset of patients [4,5] and highlight the importance of VTE preventions in COVID-19 patients in view of the worst prognosis.

In the last year, thromboembolic complications in patients receiving the SARS-CoV-2 vaccine were reported [6,7]. It has been hypothesized that the SARS-CoV-2-vaccine might induce immune thrombotic thrombocytopenia (VITT) [8,9,10,11] and cerebral sinus vein thrombosis (CVT) complicated with intracerebral hemorrhage (ICH) [12]. Although rare, this complication implies potentially relevant consequences. In this Special Issue, Gessler et al. [13] proposed a treatment algorithm in case of VITT-related CVT. Particularly, authors provided neurosurgical and haematological considerations for the management of such complex patients to optimize neurosurgical care.

In a systematic review with meta-analysis, Tufano et al. [14] evaluated the risk difference (RD) of the occurrence of VTE, Pulmonary Embolism (PE) and Deep Vein Thrombosis (DVT) between COVID-19 cohorts and other pulmonary infection cohorts, particularly with influenza A (H1N1), and in an Intensive Care Unit (ICU) setting. Notably, they observed a 6% increased risk for VTE in COVID-19 patients as compared with non-COVID-19 patients, especially in patients admitted to the ICU. These results remarked the growing burden of evidence that reported a high prevalence of VTE events in COVID-19 patients, with an increased risk in the most critical ones [15,16].

In common clinical practice, D-dimer is routinely used in the diagnostic scoring systems to rule out PE [17]. However, D-dimer specificity decreases as the age of the patient increases, and the plasma levels of D-dimer increase during inflammation and infection; therefore, the use of this parameter is cumbersome in such clinical settings. Quezada-Feijoo and co-workers [18] evaluated the diagnostic accuracy and reproducibility of the Wells and Geneva clinical probability scales and their association with D-dimer in the diagnosis of PE in elderly patients with COVID-19. The group identified a D-dimer cut-off point of >4.33 mg/L that could discriminate false positives with a specificity of 93.9%; therefore, an increased D-dimer value and clinical scales can help improve the diagnosis of PE in the elderly population.

Lastly, Mangiafico and co-workers [19] reviewed the pathophysiology of vascular damage and the hypercoagulative state related to SARS-CoV-2 infection and the role of heparin in various COVID-19 clinical settings according to the most recent evidence [20,21,22,23,24]. Authors concluded that the prophylactic dose of heparin is recommended in all hospitalized patients unless contraindicated, while the therapeutic dose could be considered in non-pregnant patients requiring low-flow supplemental oxygen, with increased D-dimer levels and low bleeding risk [25,26,27]. Prophylactic heparin could also have a role in the prevention of postdischarge COVID-19 sequelae, in the presence of high-risk clinical features that raise the risk of thrombotic complications [28,29].

Although COVID-19 continues to be a global emergency, great efforts have been made over the past two years to improve the prognosis of patients. Certainly, the papers published in this Special Issue contribute to the growing scientific evidence concerning the pathogenesis and treatment of thromboembolic complications in COVID-19. Therefore, my gratitude goes to the authors, the reviewers and the editorial office for the excellent work done.
